# Mapping oral-systemic health relationships: a data-driven analysis of co-occurrence patterns in biomedical literature

**DOI:** 10.1093/jamiaopen/ooag130

**Published:** 2026-07-10

**Authors:** Bhumi Patel, Janusz Wojtusiak

**Affiliations:** Department of Health Administration and Policy, College of Public Health, George Mason University, Fairfax, VA 22030, United States; Department of Health Administration and Policy, College of Public Health, George Mason University, Fairfax, VA 22030, United States

**Keywords:** cluster analysis, oral health, comorbidity, bibliometrics, machine learning

## Abstract

**Objective:**

To systematically map co-occurrence patterns between systemic medical conditions and oral diseases in the biomedical literature.

**Materials and Methods:**

We conducted a large-scale bibliometric analysis of PubMed-indexed literature from 1995 to 2025. Using automated Application Programming Interface queries, we retrieved publications jointly referencing 256 systemic conditions (defined using the Agency for Healthcare Research and Quality’s Clinical Classifications Software) and 5 oral health conditions (periodontal disease, dental caries, tooth loss, oral ulcers, and oral cancers). Search strategies combined Medical Subject Headings and title/abstract terms. Co-occurrence data were normalized using max scaling and analyzed using hierarchical clustering, K-means clustering, and network visualizations. This study reports associations between conditions and not causal relationships.

**Results:**

The search identified 79 817 co-mention instances across 39 782 unique publications. Of 256 medical conditions, 237 co-occurred with at least one oral condition. Periodontal diseases and oral cancers accounted for the largest share of co-mentions (42 554 and 38 735, respectively). Hierarchical clustering of systemic conditions revealed eight clusters, including malignancies strongly co-occurring with oral cancers and immunity disorders co-occurring with periodontal diseases. Dental conditions grouped into three clusters: (1) caries, ulcers, and tooth loss; (2) oral cancers; and (3) periodontal diseases. Publication-level clustering identified 6 thematic domains dominated by oncology and inflammation research.

**Conclusion:**

This study provides a data-driven overview of oral-systemic health research across three decades, highlighting well-studied co-occurrence patterns, such as periodontal-diabetes, oral cancer-malignancies and less frequently represented areas involving caries, ulcers, and tooth loss, underscoring opportunities for interdisciplinary research and integrated care models.

## Introduction

In the vast landscape of human health, the body’s systems function as interconnected networks rather than isolated units. One such connection that has garnered growing attention in clinical and research communities is the link between systemic and oral health. While this relationship may appear modern, it has historical roots dating back to ancient civilizations, in which rudimentary oral hygiene practices reflected an early awareness of oral health’s relevance to general well-being. Scientific understanding solidified in the 20th century with the focal infection theory, which proposed that chronic oral infections could contribute to systemic disease through microbial dissemination and inflammation.^1^^,^^2^

Today, this concept has evolved under scientific scrutiny and aligns with current biological models. Research suggests that inflammatory mediators from periodontal disease can enter the systemic circulation, may contribute to chronic inflammation and increasing susceptibility to conditions such as diabetes, cardiovascular disease, and rheumatoid arthritis.^3^^,^^4^ For instance, *Porphyromonas gingivalis*, a key periodontal pathogen, has been studied in relation to vascular changes in cardiovascular disease and identified in the synovial fluid of patients with rheumatoid arthritis, suggesting a potential etiological role.^3^^,^^5^ The relationship between periodontitis and diabetes is particularly well-established and recognized as bidirectional: diabetes elevates the risk of periodontal disease, while periodontal inflammation can worsen glycemic control.^6^^,^^7^ Beyond periodontitis, other prevalent oral diseases including dental caries, tooth loss, oral ulcers, and oral cancers are increasingly recognized as part of the broader oral-systemic health landscape. These conditions are associated with a range of systemic states including immune dysfunction, nutritional deficiencies, viral oncogenesis, and chronic inflammation, yet they remain far less studied than periodontal disease in the context of systemic health.

Non-communicable diseases—such as cardiovascular, respiratory, kidney, and liver diseases, as well as cancers and metabolic disorders—account for approximately 74% of all global deaths.^7^ Periodontitis itself ranks among the most prevalent chronic inflammatory diseases, affecting an estimated 19% of adults worldwide.^8^ As these conditions increase in frequency, often coexisting within individuals, the prevalence of multimorbidity has risen sharply, now impacting more than 20% of the global adult population.^9^ These patterns underscore the urgent need for a comprehensive understanding of how systemic and oral conditions interact and how these interactions are represented in scientific research across disciplines. Recent reviews further document the broad pathogenic links between periodontal disease and systemic health, covering shared biomarkers, diagnostic innovations, and cardiovascular implications, reinforcing the clinical rationale for examining oral-systemic relationships at scale.

Although the literature linking oral and systemic health has expanded significantly, most existing research focuses on specific disease pairings, such as diabetes and periodontitis or cardiovascular disease and oral inflammation.^10^ While some studies explore broader systemic implications, they often limit their oral health perspective primarily to periodontitis.^11^^,^^12^ Consequently, other prevalent oral diseases like dental caries, tooth loss, oral ulcers, and oral cancers are rarely examined concurrently alongside a wide array of systemic conditions, leaving significant gaps in our understanding of the full spectrum of oral-systemic interrelationships. This limitation reflects not only a gap in clinical evidence but also a gap in how these relationships are structured and represented in biomedical research publications.

This limitation highlights the need for a comprehensive, large-scale assessment capable of simultaneously considering numerous systemic and dental conditions. Such an analysis is crucial for mapping the broader patterns of their co-occurrence within the scientific literature. It is important to note, however, that patterns of co-occurrence in biomedical publications reflect research attention and thematic organization within the literature, rather than causal, biological, or clinical relationships between conditions. Understanding these patterns can reveal which medical-dental combinations are heavily studied vs overlooked, uncover clusters of conditions frequently investigated together, guide future interdisciplinary research, and inform integrated care models responsive to real-world multimorbidity. Addressing this limitation requires computational approaches capable of handling the vast scale and complexity of the biomedical literature.

In this study, we address the identified gaps by systematically mapping co-occurrence patterns between a wide range of systemic medical conditions and multiple oral diseases within the biomedical literature, in order to characterize the thematic structure of oral-systemic health research. This research leverages computational methods to retrieve and cluster thousands of publications indexed in PubMed. Our objectives are to: (1) report on the number of publications that include any combination of medical and dental conditions; (2) identify clusters of medical conditions measured by their co-occurrence in published literature in relation to dental conditions; (3) identify clusters of dental conditions by their co-occurrence in published literature in relation to medical conditions; and (4) identify clusters of publications based on common medical and dental conditions. Through this data-driven mapping approach, we provide a novel, comprehensive perspective on how oral and systemic health interrelationships are currently represented in scientific research, revealing both well-explored and underexplored areas across multiple oral conditions, and thereby guiding future research toward integrated health care strategies.

## Methods

This study employed a data-driven approach to analyze co-occurrence patterns between systemic medical conditions and oral diseases in the biomedical literature indexed in PubMed. Unlike systematic reviews that focus on evidence synthesis for specific associations, our methodology aimed to identify broader patterns and clusters of conditions based on their co-occurrence frequency in the published literature. The overall framework of this research is depicted in Figure S1 in the Supplementary Material.

### Data source and retrieval

The PubMed database, maintained by the U.S. National Library of Medicine, was our primary data source due to its comprehensive coverage of biomedical literature. We systematically retrieved publication records using the PubMed Application Programming Interface (API), facilitating automated querying based on predefined criteria. Publications indexed between January 1995 and March 2025 were included. Retrieval was restricted to human studies published in English with available abstracts, which is consistent with standard practice in large-scale bibliometric analyses, though it may limit the representation of non-English literature.

Systemic medical conditions were defined according to the Agency for Healthcare Research and Quality’s (AHRQ) Clinical Classifications Software (CCS), which condenses thousands of International Classification of Diseases (ICD) codes into 258 clinically meaningful single-level diagnostic categories.^13^ Of the 258 CCS categories, 2 (*Disorders of teeth and jaw* and *Secondary malignancies*) were excluded prior to analysis because they directly overlap with or subsume the oral health conditions under study, which would introduce circularity in co-occurrence measurement. The remaining 256 CCS categories were used as the operational set of systemic medical conditions throughout all analyses. This categorization allows manageable yet comprehensive analysis of condition patterns in large bibliometric datasets. Five prevalent oral health conditions known to be associated with systemic diseases were selected: periodontal diseases, dental caries, tooth loss, oral ulcers, and oral cancers.

### Literature search strategy

Separate search queries were developed for each medical and dental condition using Medical Subject Headings (MeSH terms) and relevant keywords found in article titles and abstracts. MeSH terms were carefully selected and validated using the NCBI MeSH Browser to ensure accurate and comprehensive retrieval. The final search process employed an iterative computational approach, where each medical condition query was systematically combined with each dental condition query through an automated iterative search process through PubMed’s API.

The general structure of a query was:

(medical condition [MeSH] OR medical condition [Title/Abstract]) AND

(dental condition [MeSH] OR dental condition [Title/Abstract]) AND

(1995:2025[PDAT]) AND (english[lang]) AND (hasabstract) AND (Humans[MeSH])

This structured and automated method facilitated comprehensive and reproducible retrieval of publications documenting co-occurrences. The primary output of this stage was a dataset linking PubMed Identifiers (PMIDs) to the co-occurring medical and dental terms identified within each publication.

### Data analysis

The retrieved dataset, linking PMIDs to medical and dental conditions, was processed to facilitate multiple analytical goals described below.

### Data processing and normalization

For hierarchical clustering of conditions (Goals 2 and 3), the co-occurrence matrix was z-score standardized (mean = 0, unit variance) to reduce the influence of highly frequent conditions and emphasizes relative co-occurrence patterns.

To improve interpretability of results, in heatmap visualizations (Figures S2-S4), co-occurrence counts were max-scaled within each column to improve visual comparability across conditions. In contrast, the publication-level clustering (Goal 4) utilized a raw binary matrix (mention vs no-mention), as this format is most appropriate for the Jaccard distance metric employed during dimensionality reduction.

#### Descriptive analyses (Goal 1)

Basic descriptive statistics were calculated using the initial dataset, including the total number of unique publications retrieved that linked any specified medical condition with any specified dental term.

#### Co-occurrence matrices for association analysis (Goals 2 and 3)

To identify clusters of medical and dental conditions based on their co-occurrence in the literature, we first created a co-occurrence matrix for each goal. Raw frequency counts were used because they reflect the actual volume of research attention received by specific condition pairs, making them well-suited for measuring relative co-occurrence intensity across the literature. Total co-occurrence counts were derived by summing across rows and columns to describe overall co-occurrence frequency. Hierarchical clustering was applied to group conditions based on their co-occurrence patterns using Ward’s linkage method with Euclidean distance applied to *z*-score standardized matrices. This method was selected because it reveals nested groupings without requiring a predefined number of clusters, making it well-suited for exploratory pattern detection in co-occurrence data where condition relationships are not known a priori. Dendrogram cutoff thresholds were determined through an iterative evaluation process in which multiple distance thresholds were tested, and the resulting cluster solutions were assessed for thematic interpretability, within-cluster homogeneity, and between-cluster separation. Clustering robustness was evaluated using the cophenetic correlation coefficient, which measures how faithfully the dendrogram preserves the original pairwise distance structure, and silhouette scores, which assess cluster compactness and separation.

For Goal 2 (medical-condition clustering), a threshold of 7.0 was applied, producing eight distinct clusters. This threshold yielded a silhouette score of 0.655, among the highest observed across the tested range (thresholds 5.0-10.0; silhouette scores 0.471-0.665), while producing the most thematically distinct and interpretable groupings. Although a threshold of 9.0 produced a marginally higher silhouette score (0.665), it resulted in the merging of thematically distinct condition groups into 7 clusters, reducing thematic resolution. The cophenetic correlation coefficient of 0.844 confirmed strong preservation of the original distance structure.

For Goal 3 (dental-condition clustering), a threshold of 25.0 was applied, producing three distinct clusters. The cophenetic correlation coefficient of 0.974 indicated near-perfect preservation of the original pairwise distance structure. The silhouette score of 0.223 reflects the inherently limited statistical power of clustering 5 observations into three groups; however, the sensitivity analysis demonstrated that the three-cluster solution was highly stable across a wide range of thresholds (18.0-28.0), with all values producing identical cluster assignments and silhouette scores. A reduction to 2 clusters at threshold 30.0 yielded only a marginal improvement in silhouette score (0.234) at the cost of merging thematically distinct dental conditions. The three-cluster solution was, therefore, retained as the most interpretable and stable configuration.

#### Binary matrix for publication clustering (Goal 4)

To cluster publications based on shared medical and dental condition profiles, a binary feature matrix was constructed in which each row represented a unique PMID, and each column represented a medical or dental condition. K-Means clustering was selected because it is computationally efficient and suitable for partitioning low-dimensional embeddings into distinct groups based on shared co-occurrence profiles. To reduce dimensionality while preserving similarity structure, Uniform Manifold Approximation and Projection (UMAP) was applied to the binary matrix using 2 dimensions and Jaccard distance. K-means clustering was then performed on the 2-dimensional UMAP embedding to assign each publication to a cluster.

The number of clusters was evaluated using inertia and silhouette analysis across k=2-10. The selected solution of k=6 produced a silhouette score of 0.361 and corresponded to a clear elbow in the inertia curve, while also yielding thematically coherent publication groups. Although k=7 produced a slightly higher silhouette score, the k=6 solution was retained because it provided a more parsimonious and interpretable thematic organization.

#### Visualization

Visualizations included dendrograms, heatmaps, and network graphs to summarize condition and publication clusters.

## Results

An automated search of PubMed to identify publications, from January 1995 to March 2025 that reported co-occurrences between 256 medical CCS Categories and 5 specified dental conditions was conducted. This search yielded 79 817 co-mention instances across 39 782 unique PubMed-indexed publications. Among the 256 medical conditions searched, 237 were found to co-occur with at least one of the dental conditions, providing a comprehensive dataset for subsequent clustering and pattern analysis. The frequency counts derived from the 79 817 instances were used for analyzing association patterns and clustering conditions (Goals 2 and 3), while the clustering of publications (Goal 4) utilized the 39 782 unique PMIDs.

Analysis of co-occurrence frequencies revealed significant variability. Among medical conditions, metabolic disorders, neurogenerative disorders, and malignancies exhibited the highest overall co-occurrence counts across the 5 dental conditions (Figure S2). Among the dental conditions, periodontal diseases showed the highest overall co-occurrence count across the medical conditions (Figure S3). These frequencies indicate varying levels of representation for different systemic-oral condition pairs within the literature corpus.

### Clustering of medical conditions (Goal 2 analysis)

**Figure 1.**
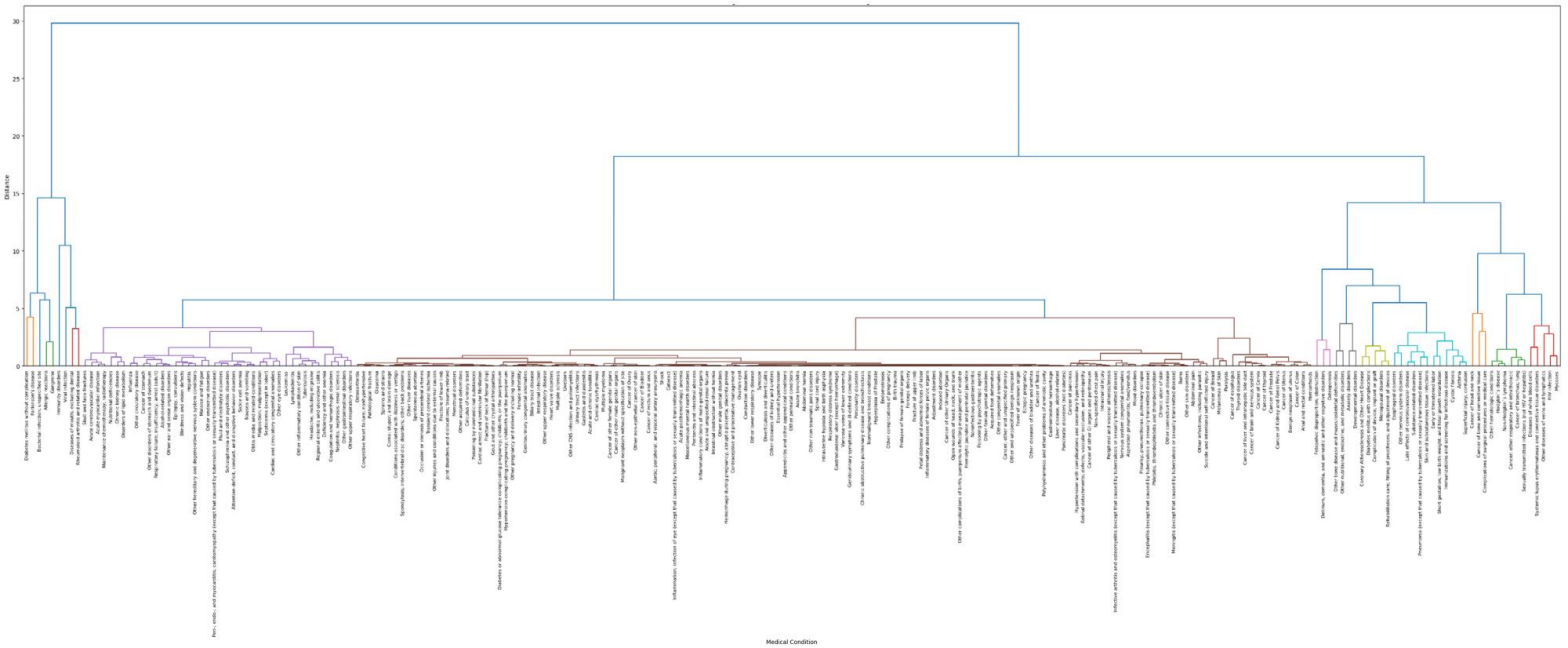
Hierarchical clustering of medical conditions. Dendrogram displaying hierarchical clustering of 237 systemic medical conditions based on their co-occurrence patterns with 5 dental conditions. Ward’s linkage method with Euclidean distance was applied to standardized co-occurrence data. The distance threshold of 7.0 (horizontal line) defines eight distinct clusters, each represented by a different color. The hierarchical structure reveals natural groupings of medical conditions that exhibit similar patterns of co-occurrence with oral health conditions in the published literature.

**Figure 2.**
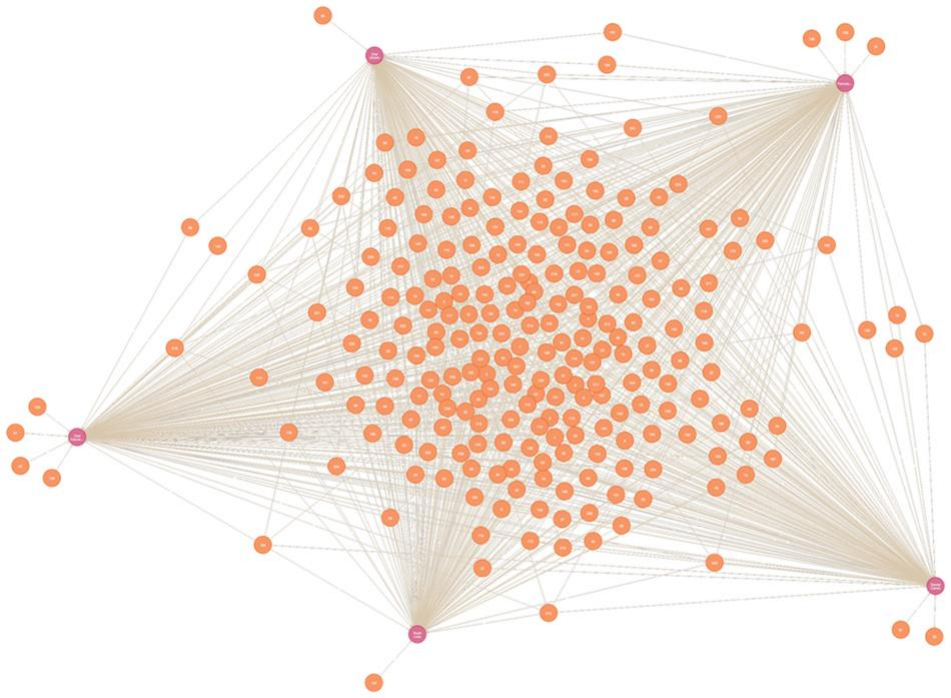
Network graph of medical-dental conditions. Bipartite network connecting 237 systemic medical conditions (orange nodes) to 5 oral health conditions (pink nodes) based on literature co-mention frequency in PubMed (1995-2025). Edge weight reflects co-occurrence count in the published literature. Periodontal diseases and oral cancers show the highest node connectivity, indicating their broad co-occurrence across diverse systemic disease categories.

Clustering of the 237 × 5 co-occurrence matrix identified systemic medical conditions with similar co-occurrence patterns across oral diseases in the biomedical literature. The hierarchical clustering produced eight distinct clusters of medical conditions. The dendrogram (Figure 1) visualizes the hierarchical structure of these co-occurrence patterns, while the heatmap (Figure S2) illustrates the max-scaled co-occurrence intensity between medical and dental conditions across clusters. A medical-dental network graph (Figure 2) further highlights the interconnections between conditions.

Each cluster exhibited distinct co-occurrence patterns with the 5 dental conditions. Cluster 1 primarily included *Parkinson’s disease*, *bacterial infections*, and *diabetes mellitus without complication*. These conditions co-occurred most strongly with periodontal diseases (*n* = 10 693), followed by oral cancers (*n* = 1780). Cluster 2 encompassed *viral infections*, *rheumatoid arthritis*, and *diseases of the mouth (non-dental)*. This cluster frequently co-occurred with oral cancers (*n* = 2751), and periodontal diseases (*n* = 2087). Cluster 3 consisted solely of immunity disorders, which showed a distinct pattern of co-occurrence dominated by periodontal diseases (*n* = 1960), followed by oral cancers (*n* = 1467). Cluster 4 included *breast cancer*, *melanoma*, *leukemias*, *chronic kidney disease*, and *lipid metabolism disorders*. This cluster exhibited strong co-occurrence with oral cancers (*n* = 7844) and periodontal diseases (*n* = 6925). Cluster 5 represented *obstetric complications*, *dementia and cognitive disorders*, and *osteoporosis*. Co-occurrence was moderate across all dental conditions, with highest frequencies seen for periodontal diseases (*n* = 1315) and tooth loss (*n* = 511). Cluster 6 included *developmental disorders*, *coronary atherosclerosis*, and *diabetes with complications*. This cluster demonstrated high co-occurrence with periodontal diseases (*n* = 6696), followed by dental caries (*n* = 2697). Cluster 7, focused *head and neck cancers*, *surgical complications*, and *bone/connective tissue cancers*, showed a concentrated co-occurrence pattern with oral cancers (*n* = 5812) and periodontal diseases (*n* = 1879). Finally, Cluster 8 was composed mainly of *non-Hodgkin’s lymphoma*, *HIV infection*, *lung cancer*, and *Hodgkin’s disease*. This cluster had the highest co-occurrence with oral cancers (*n* = 7221), and periodontal diseases (*n* = 2002).

A summary of the top medical and dental conditions per cluster is provided in Table S1 (see Supplementary Material). Collectively, these results reveal the structural patterns of how medical conditions group based on their co-occurrence with dental conditions in PubMed-indexed literature.

Figure S2 presents a heatmap of the top medical conditions in each cluster, showing their max-scaled co-occurrence intensity with 5 key oral health conditions (see Supplementary Material). Stronger associations (darker shades) highlight specific systemic diseases that tend to co-occur more frequently with particular dental conditions, such as *Parkinson’s disease* and *periodontal diseases*, or *head and neck cancers* and *oral cancers*. This visual emphasizes the heterogeneity in medical-dental relationships and how certain disease groups concentrate around particular oral health themes. These patterns reflect the volume of co-occurrence in the biomedical literature and do not indicate causal or clinical relationships.

Figure 2 illustrates a bipartite network graph connecting systemic medical conditions (orange nodes) with oral health conditions (pink nodes) based on their co-occurrence in the literature. Edge density and connectivity reveal the extent to which different systemic diseases are linked to multiple oral conditions. The network structure underscores the central role of periodontal diseases and oral cancers, which connect broadly across numerous systemic categories, reflecting their frequent mention in multidisciplinary health research.

### Clustering of dental conditions (Goal 3 analysis)

To examine how dental conditions group based on their co-occurrence patterns with systemic medical conditions, we conducted hierarchical clustering using a standardized dental × medical co-occurrence matrix. This matrix reflected the frequency with which each of the 5 dental conditions—periodontal diseases, dental caries, tooth loss, oral ulcers, and oral cancers—co-occurred with 237 medical conditions in PubMed-indexed publications.

**Figure 3.**
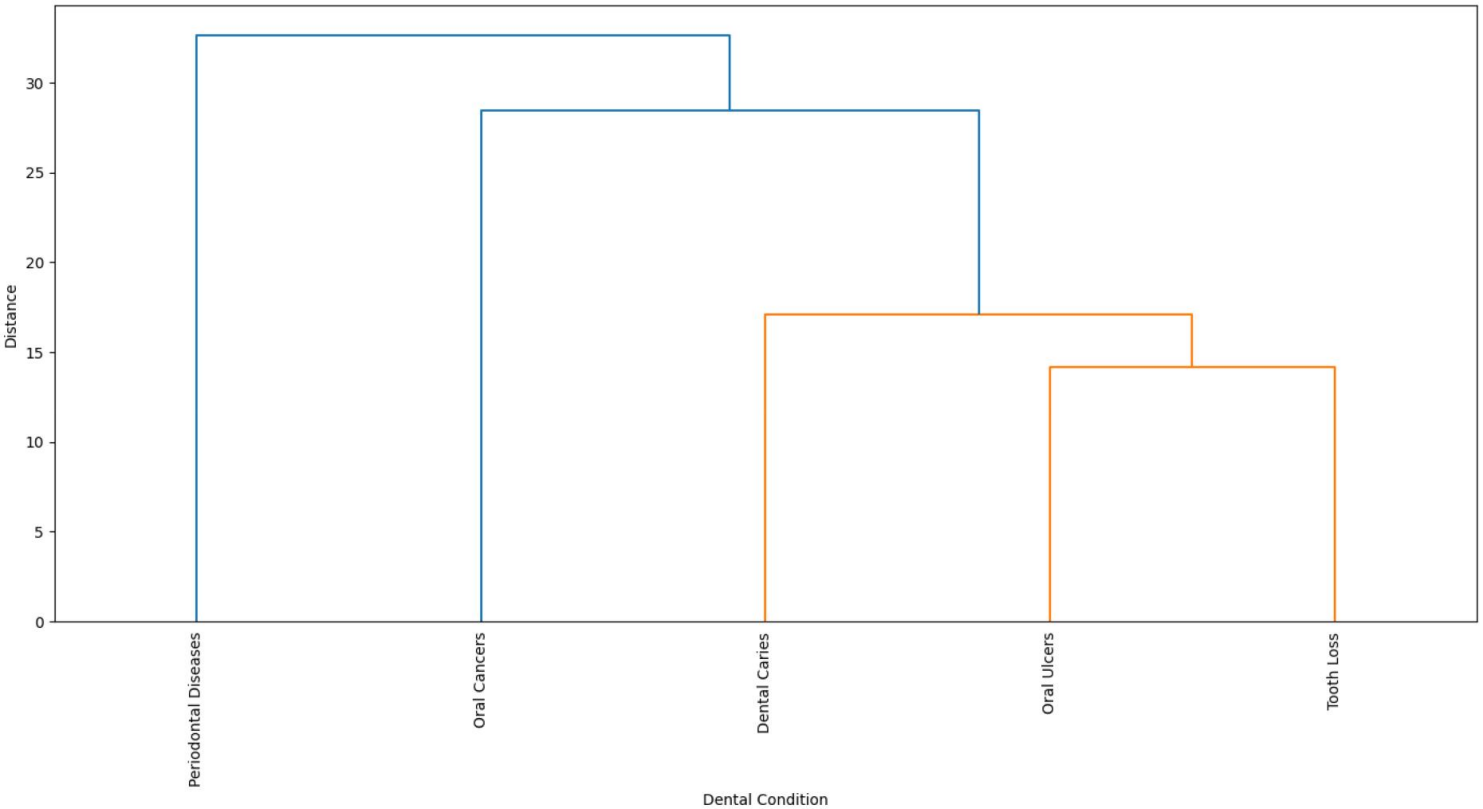
Hierarchical clustering of dental conditions. Dendrogram illustrating hierarchical clustering of 5 dental conditions based on co-occurrence patterns with 237 systemic medical conditions retained from the PubMed search. The co-occurrence matrix was transposed and standardized prior to clustering. Ward’s method with a distance threshold of 25.0 defines three distinct clusters: (1) dental caries, oral ulcers, and tooth loss; (2) oral cancers; and (3) periodontal diseases. The hierarchical structure reflects distinct research themes and systemic disease co-occurrence profiles for dental condition cluster.

**Figure 4.**
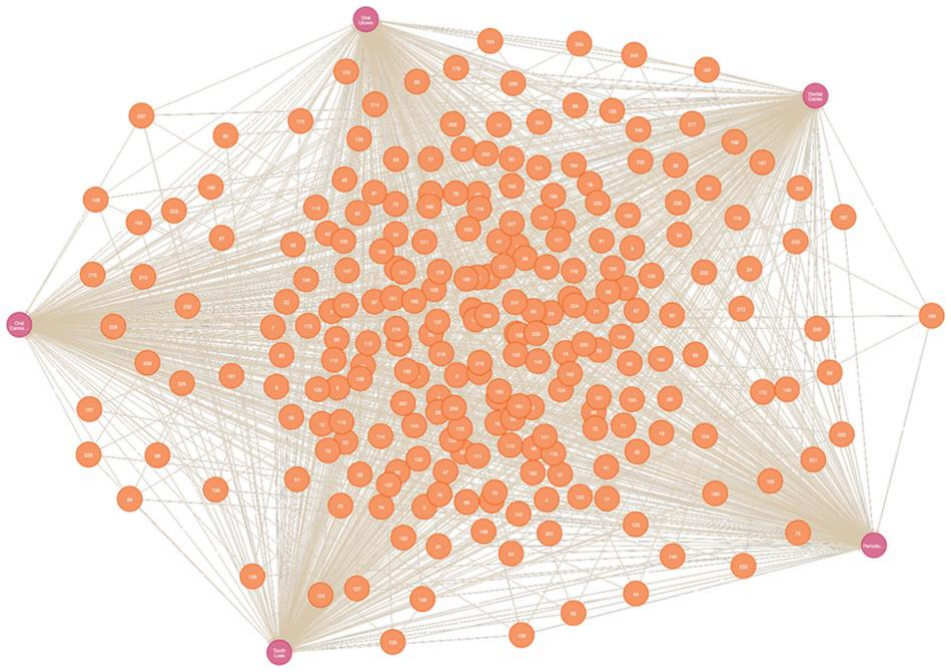
Network graph of dental-medical conditions. Network illustrating co-occurrence patterns between the 5 dental conditions (central nodes) and systemic medical conditions (peripheral nodes), weighted by shared publication frequency. Periodontal diseases and oral cancers display the densest connectivity, while dental caries, oral ulcers, and tooth loss show comparatively sparse co-occurrence patterns reflecting their lower representation in multi-systemic oral health research.

The resulting dendrogram (Figure 3) revealed three distinct clusters, which were then further examined using a heatmap (Figure S3) and a medical-dental network graph (Figure 4).

Cluster 1 included dental caries (*N* = 8220), oral ulcers (*N* = 4921), and tooth loss (*N* = 3703), which tended to co-occur with a broad range of systemic diseases, particularly immune-related, infectious, and developmental conditions. The most frequently co-occurring medical conditions were *Immunity disorders* (*n* = 1364), *Bacterial infection; unspecified site* (*n* = 698), and *Viral infection* (*n* = 695).

Cluster 2 consisted solely of oral cancers, reflecting a highly focused research domain. The most frequently co-occurring medical conditions in this cluster were *Cancer of head and neck* (*n* = 2626), *Cancer of bone and connective tissue* (*n* = 1848), and *Viral infection* (*n* = 1739). Oral cancers demonstrated 29 416 total co-occurrences with systemic conditions, reflecting their centrality in cancer-focused oral-systemic co-occurrence patterns.

Cluster 3 contained periodontal diseases as the sole condition and showed the broadest and strongest co-occurrence patterns. The top co-occurring conditions included *Parkinson’s disease* (*n* = 3231), *Bacterial infection; unspecified site* (*n* = 2218), and *Diabetes mellitus without complication* (*n* = 1978). Other notable co-occurrences included gangrene, surgical complications, rheumatoid arthritis, and viral infection. Periodontal diseases accounted for 33 557 total co-occurrences, making it the most prevalent dental condition in systemic co-mention analysis. A detailed summary of the top dental-medical co-occurrence counts within each cluster is provided in Table S2 (see Supplementary Material).

Figure S3 displays a heatmap illustrating the top systemic medical conditions co-occurring with each dental condition cluster (see Supplementary Material). Higher-intensity shading highlights clusters of research interest, showing clear concentration patterns—for example, stronger co-occurrence between periodontal diseases and metabolic/immune-related conditions, and between oral cancers and oncologic systems. These patterns reflect literature co-occurrence frequency and do not imply causal relationships.

Figure 4 depicts a dental-medical co-occurrence network, where dental conditions (central nodes) are connected to medical conditions (peripheral nodes) based on literature frequency. Dense interconnectivity around periodontal diseases and oral cancers demonstrates their high research prominence and broad systemic relevance. In contrast, nodes representing dental caries, oral ulcers, and tooth loss show more distributed, moderate-strength co-occurrence patterns, aligning with the cluster analysis.

### Clustering of publications (Goal 4 analysis)

**Figure 5.**
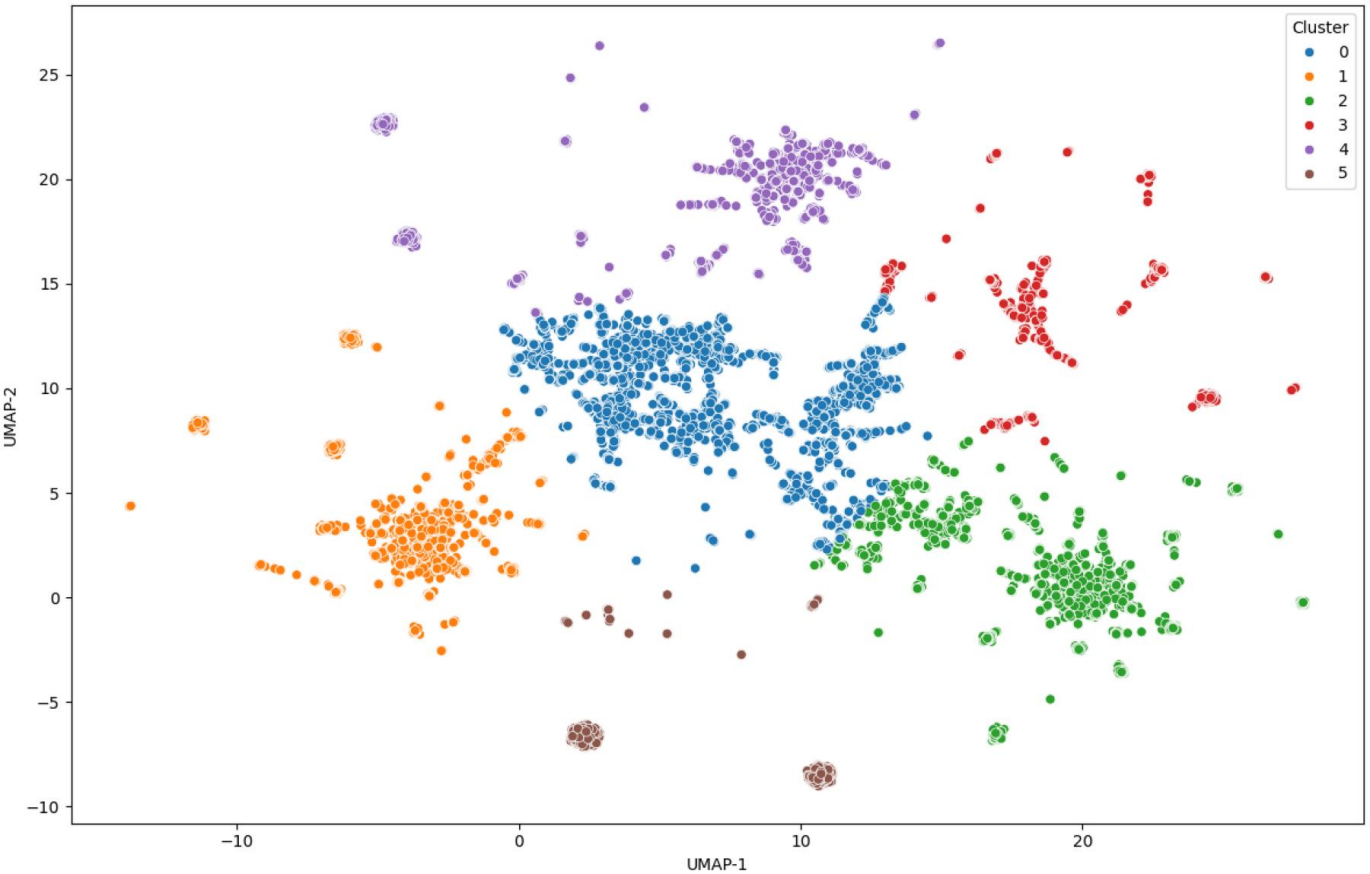
K-means clustering of publications. UMAP visualization of PubMed publications clustered into 6 thematic domains using K-means clustering based on medical-dental condition co-occurrence profiles. Each point represents a unique publication; colors indicate cluster assignment. Separation between clusters suggests distinct thematic organization around major oral-systemic health research domains. Clusters reflect shared publication co-occurrence patterns only and do not indicate clinical or causal associations between conditions.

The UMAP projection (Figure 5) showed separation between 6 publication clusters with minimal overlap.

Cluster 0 was characterized by oral-systemic inflammatory and infectious topics, with *Periodontal diseases* (*N* = 6258), *Tooth loss* (*N* = 2189), *Immunity disorders* (*N* = 1970), and *Oral ulcers* (*N* = 1652) among the most frequent conditions. Cluster 1 centered on pregnancy, developmental disorders, and skeletal/muscular topics, with *Periodontal diseases* (*N* = 7571), *Diabetes mellitus without complication* (*N* = 835), and *Gangrene* (*N* = 759) among the leading conditions.

Cluster 2 was dominated by cancer-focused publications, particularly *Oral cancers* (*N* = 8576), *Surgical complications* (*N* = 1048), *Respiratory/intrathoracic cancers* (*N* = 896), and *Cancer of head and neck* (*N* = 742). Cluster 3 emphasized immune system dysfunction, hematologic diseases, and viral conditions, including *Oral cancers* (*N* = 3046), *Viral infections* (*N* = 1829), *Immunity disorders* (*N* = 1413), *Non-Hodgkin’s lymphoma* (*N* = 1011), and *Hodgkin’s disease* (*N* = 992).

Cluster 4 included literature focused on chronic systemic disease, mental health, and oral decay. The most frequently mentioned conditions included *Dental caries* (*N* = 3510), *Periodontal diseases* (*N* = 3289), *Bacterial infection; unspecified site* (*N* = 1544), and *Allergic reactions* (*N* = 1379). Cluster 5 focused on neurodegenerative and comorbid systemic disorder. Leading co-occurring conditions were *periodontal diseases* (*N* = 2114), *Parkinson’s disease* (*N* = 2055), *Oral cancers* (*N* = 1859), and *Cancer of head and neck* (*N* = 1644) as the leading conditions.

A comprehensive overview of the top medical and dental condition co-occurrence counts within each publication-derived cluster is provided in Table S3 (see Supplementary Material).

Figure S4 presents a heatmap of the top conditions by publication cluster, displaying their co-occurrence counts (see Supplementary Material). Each row corresponds to a publication cluster, while each column shows a medical or dental condition. The visualization emphasizes thematic concentration—eg, clusters 2 and 3 are dominated by oncology and immune-related conditions, respectively, while cluster 0 exhibits a more distributed co-occurrence across inflammatory and dental diseases. This heatmap provides a quantitative profile of the thematic composition of each publication group.

**Figure 6.**
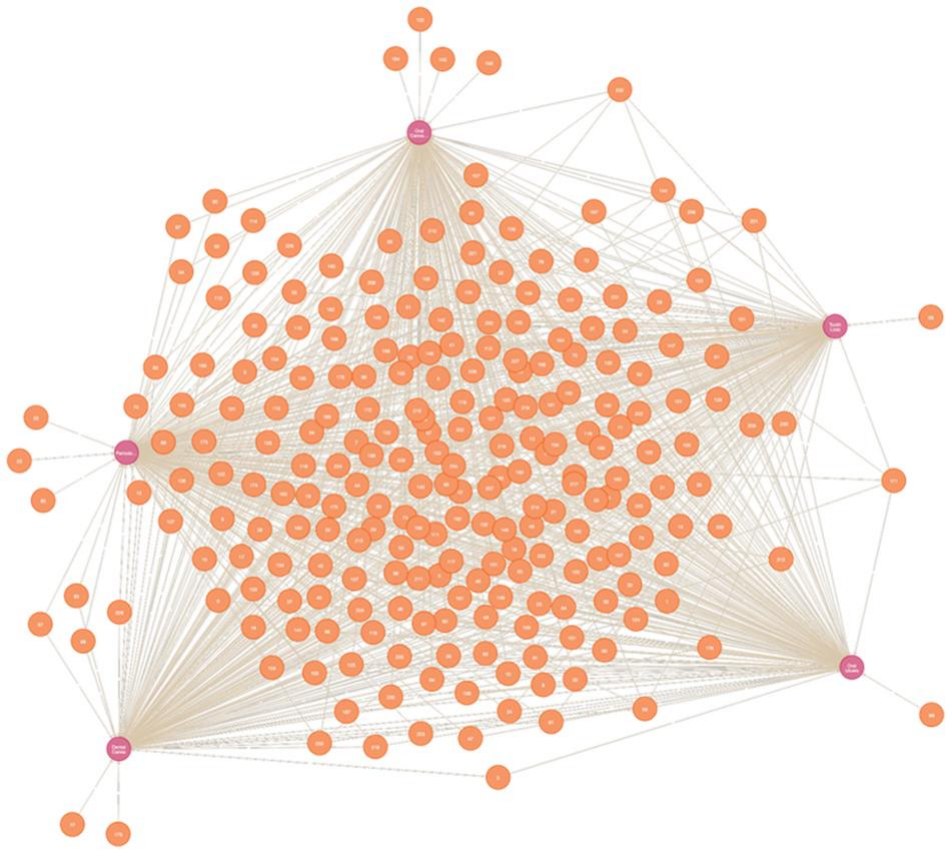
Network graph of publication clusters vs conditions. Condition-level network connecting medical and dental conditions (peripheral nodes) to their corresponding publication clusters (central nodes), weighted by co-mention frequency across 39 782 publications. Periodontal diseases, oral cancers, and dental caries serve as major cross-cluster hubs, while cluster-specific conditions (eg, Parkinson’s disease in Cluster 5; head and neck cancers in Cluster 2) reflect thematic specialization within publication groups.

Figure 6 displays the condition-level network graph, connecting medical and dental conditions based on shared co-mention in clustered publications. Dental nodes are centrally located, with periodontal diseases, oral cancers, and dental caries acting as major hubs. Peripheral medical conditions show variable connectivity depending on thematic overlap. This graph offers a holistic view of how specific medical and dental terms are interconnected in the literature and reinforces the centrality of certain oral conditions in multidisciplinary biomedical research.

## Discussion

This study employed a large-scale, data-driven bibliometric approach to map co-occurrence patterns. Across 79 817 co-mention instances spanning 39 782 publications, we identified medical conditions with prominent oral co-occurrence patterns (eg, periodontal diseases, viral and immunity disorders, oncologic conditions), clusters of systemic conditions sharing similar dental-co-occurrence profiles, clusters of dental conditions grouped by systemic disease patterns, and thematic clusters of publications. Together, these findings provide a quantitative literature-level landscape of oral-systemic health research, revealing structured research domains across decades of biomedical scholarship.

### Interpretation of medical condition clusters (Goal 2)

The hierarchical clustering of systemic conditions based on their co-occurrence patterns with dental conditions revealed eight distinct clusters, reflecting both broad and highly focused oral-systemic co-occurrences. These groupings illustrate how specific systemic diseases cluster with particular dental conditions in the biomedical literature, with periodontal diseases emerging as the most frequently co-mentioned oral condition across diverse systemic categories.

Cluster 1, the largest and most heterogeneous group in terms of co-occurrence volume, included conditions such as *Parkinson’s disease*, *bacterial infections*, and *diabetes mellitus without complications*, which most frequently co-occurred with periodontal diseases. The co-mention of Parkinson’s disease and periodontal diseases in the literature is consistent with emerging research suggesting shared inflammatory and neurological mechanisms described in prior studies of these topics.^4^^,^^14^ The co-occurrence with diabetes aligns the well-documented bidirectional research pattern described in prior literature, wherein diabetes increases periodontal susceptibility, and periodontal inflammation may impair glycemic control.^15–17^ Cluster 2 featured viral and autoimmune conditions, including *HIV*, *viral infections*, and *rheumatoid arthritis*, showing strong co-occurrence with oral cancers and periodontal diseases. These findings are consistent with prior literature describing co-occurrence patterns involving systemic immune dysfunction, oral mucosal conditions, and oral cancer risk.^18^ Cluster 3, composed solely of immunity disorders, exhibited prominent co-mention with periodontal diseases and oral cancers. This aligns with prior research literature at the intersection of immunological conditions, periodontal disease, and oral carcinogenesis.^19^ Cluster 4 contained solid tumors and procedural complications, including *breast cancer*, *melanoma*, and *paralysis*, and showed high co-occurrence with oral cancers and periodontal diseases. This research volume likely reflects the extensive literature on oral complications of cancer treatment including mucositis, xerostomia, and opportunistic infections rather than a direct oncologic relationship.^20^^,^^21^ Cluster 5 included obstetric complications, cognitive disorders, and osteoporosis, with moderate co-mention across periodontal diseases and tooth loss. These co-occurrence patterns may reflect systemic aging, nutritional deficiencies, or physical limitations influencing oral hygiene and disease progression. Cluster 6, a mix of neurologic and endocrine conditions, exhibited broad co-mentions across multiple dental conditions, suggesting systemic frailty or chronic disease burden as a possible context of these patterns in the literature. Cluster 7, focused on head and neck cancers and surgical complications, showed a highly specific pattern of co-occurrence with oral cancers, underscoring a well-defined literature focused on oncology and oral pathology.^20^^,^^21^ Finally, Cluster 8 composed of hematologic and respiratory malignancies (eg, *non-Hodgkin’s lymphoma*, *lung cancer*) showed high co-occurrence with oral cancers and periodontal diseases, potentially due to the frequent co-mention of oral manifestations of systemic malignancies or effects of chemotherapeutic regimens in the literature.^22–24^ Overall, this clustering approach uncovered both well-established oral-systemic co-occurrence patterns and underexplored patterns of co-mention, providing a literature-level map of how systemic health conditions are represented alongside oral health topics in biomedical research.

### Interpretation of dental condition clusters (Goal 3)

Clustering the 5 dental conditions by their co-occurrence with 237 systemic medical conditions revealed three conceptually distinct groups, reflecting different thematic concentrations in the literature.

Cluster 1 grouped dental caries, oral ulcers, and tooth loss, conditions often described in the literature on compromised oral environments, poor hygiene, or systemic vulnerability. Their shared co-occurrence patterns with infections, immune disorders, and oral soft tissue diseases suggest they frequently represented in contexts of general health decline or immune dysfunction. For instance, oral ulcers are often reported alongside systemic immune or viral conditions, while caries and tooth loss are influenced by diet, salivary function, and chronic disease burden.^11^^,^^25^

Cluster 2, composed solely of oral cancers, formed a distinct cluster with frequent co-mention of head and neck cancers, surgical complications, and viral infections, including HPV. This reflects the focused nature of oral oncology research and its overlap with broader cancer and virology literature.^23^ Its separation from other dental conditions underscores its status as a specialized area within oral-systemic literature.

Cluster 3, contained only periodontal diseases, exhibited the broadest and most frequent co-occurrence patterns with systemic conditions ranging from diabetes and bacterial infections to Parkinson’s disease and cardiovascular conditions. This aligns with longstanding literature describing periodontitis in the context of chronic inflammation and systemic disease pathways, including cardiovascular, metabolic, and neurodegenerative conditions.^4^^,^^19^ The prominence of this cluster reinforces periodontal disease as a central topic in the oral-systemic literature.

Rather than interpreting this cluster as evidence that periodontal disease mediates or causes systemic outcomes, these findings should be understood as a reflection of the disproportionate research attention that periodontal disease has received across biomedical disciplines.

### Interpretation of publication clusters (Goal 4)

Clustering of publications based on co-mentioned medical and dental conditions revealed 6 thematic groups, offering insight into how oral-systemic research is organized at the study level. All 6 clusters reflect patterns of research co-mention and thematic concentration in the literature, not clinical or causal associations.

Cluster 0 was characterized by a broad distribution of oral-systemic inflammatory, infectious, and immune topics, with *Periodontal diseases, Tooth loss, Immunity disorders,* and *Oral ulcers* as the most frequently co-mentioned conditions. This cluster represents the most heterogeneous research domain, capturing publications that span multiple oral and systemic conditions simultaneously.

Cluster 1 was dominated by *Periodontal diseases* alongside obstetric, surgical, and skeletal conditions, highlights a literature subset linking periodontal disease to pregnancy outcomes, postoperative care, and musculoskeletal health. Cluster 2 captured cancer-focused publications, prominently featuring *Oral cancers*, surgical complications, and respiratory malignancies-consistent with the oncology-centered research themes identified in Cluster 7 of the medical condition analysis. Cluster 3 centered on immune dysfunction, hematologic diseases, and viral infections, with *Oral cancers*, *Viral infections*, and *Immunity disorders* most frequent—aligning with Cluster 8 of the medical condition analysis.

Cluster 4 contained literature focused on chronic systemic disease and common dental diseases, led by *Dental caries*, *Periodontal diseases*, *Bacterial infection*, and *Allergic reactions*. Cluster 5 focused on neurodegenerative and comorbid systemic disorders, with *Periodontal diseases*, *Parkinson’s disease*, *Oral cancers*, and *Cancer of head and neck* most prominent—consistent with the emerging neurodegenerative literature observed in Cluster 1 of the medical condition analysis.

Together, these clusters show that while oral cancers and periodontal diseases anchor much of the literature, significant portions of research are expanding into multisystem, age-related, and specialty-specific oral-systemic domains. The clustering structure offers a high-level map of how oral-systemic health is conceptualized in contemporary biomedical literature.

### Synthesis across analyses

The integrated findings across condition- and publication-level clustering reveal clear thematic consistencies in oral-systemic health research. Periodontal diseases and oral cancers consistently emerged as dominant topics—each forming standalone clusters in the dental condition analysis (Goal 3) and anchoring major publication clusters (Goal 4). In the medical clustering (Goal 2), systemic conditions like *immunity disorders*, *Parkinson’s disease*, *head and neck cancers*, and *hematologic malignancies* formed distinct clusters with focused co-occurrence patterns with either periodontal disease or oral cancer. The grouping of caries, oral ulcers, and tooth loss into a single dental cluster also aligned with publication clusters addressing infections, immune dysfunction, and chronic health decline, suggesting that these three conditions are less frequently studied than periodontal disease and oral cancer in the literature. Publication clusters linking oral health with pregnancy, surgical complications, cardiovascular disease, and neurodegeneration reflect distinct thematic areas within broader literature. Collectively, these analyses provide a high-level, data-driven map of how oral-systemic health is represented across biomedical literature.

### Clinical and research significance

The clinical implications of this study are indirect, as findings are derived from bibliometric analysis rather than clinical datasets. The findings carry implications for clinical research and interdisciplinary scholarship, interpreted strictly within the scope of a bibliometric analysis. Rather than directly guiding clinical screening or care protocols, this study identifies patterns of research attention, thematic organization, and gaps in the published literature—which may, in turn, inform future research priorities.

The high co-mention frequency of conditions such as periodontal disease and diabetes, or oral cancers and head and neck malignancies, reflects the continued prioritization of these topics in the research literature. This concentration of research attention is consistent with prior literature describing shared inflammatory mechanisms and bidirectional disease relationships.^26^^,^^27^ However, the co-occurrence patterns identified here are derived from bibliometric data and do not constitute independent clinical evidence for these associations.^26^^,^^27^

The relative underrepresentation of dental caries, tooth loss, and oral ulcers in multi-systemic research literature signals a meaningful gap in research attention. These conditions, though clinically prevalent and frequently co-morbid with systemic diseases, receive substantially less cross-disciplinary scholarly attention than periodontal disease or oral cancer. This observation suggests that future clinical trials, systematic reviews, and biomarker discovery studies may benefit from explicitly targeting these underexplored oral-systemic condition pairs.

The structure of publication clusters further suggests that oral-systemic research is expanding beyond traditional periodontal-diabetes pairings into domains such as neurology, oncology, obstetrics, and rheumatology. These thematic shifts in the literature may signal emerging areas of interdisciplinary scholarly interest, which could inform the design of future research programs and interdisciplinary collaborations—though validation from clinical datasets would be necessary before translating these patterns into integrated care strategies.

### Strengths and limitations

This study’s strength lies in its large-scale, automated analysis of 79 817 co-occurrence instances from PubMed, using standardized CCS codes and clustering across medical conditions, dental conditions, and publications to map the oral-systemic health research landscape.

However, several limitations must be acknowledged. Most importantly, co-occurrence in the biomedical literature does not indicate causation, directionality, or clinical significance—the clusters and associations identified here reflect how conditions are studied together in published science, not how they are mechanistically or clinically related. This analysis is also subject to publication bias, as well-funded, widely studied conditions such as periodontal disease and oral cancer will naturally dominate the co-occurrence landscape, potentially obscuring clinically meaningful but understudied associations involving dental caries, tooth loss, and oral ulcers. Restricting the search to English-language, abstract-available, human studies indexed in PubMed may further introduce selection bias, as relevant literature published in other languages or indexed in other databases (eg, Embase, Scopus, Web of Science) was not captured.

Additional methodological limitations relate to the precision of co-occurrence measurement and disease classification. Co-occurrence was assessed at the query level—a publication was counted if it matched combined MeSH and keyword queries for a given medical-dental pair—which may include some false-positive matches and may not capture studies where relationships are discussed but terminology differs from the search terms used. The CCS classification system groups thousands of ICD codes into 258 broad categories, which reduces granularity, and clinically distinct conditions sharing a CCS code are treated equivalently in this analysis. The 5 dental conditions examined, while selected for their documented systemic associations, do not represent the full spectrum of oral disease. Finally, the clustering thresholds used in Goals 2 and 3 were determined through an iterative evaluation process supported by cophenetic and silhouette analysis, though some subjectivity in final threshold selection remains inherent to exploratory bibliometric analysis.

## Conclusion

By systematically analyzing co-occurrence patterns in a large corpus of biomedical literature, this study provides a comprehensive, data-driven overview of the researched co-occurrence patterns between systemic and oral conditions. We identified distinct clusters of medical conditions, dental conditions, and publications, revealing the thematic structure, major research domains, and potential gaps within this field. Periodontal diseases and oral cancers consistently formed separate clusters, reinforcing their centrality in oral-systemic literature. Conditions such as diabetes, immunity disorders, and head and neck cancers emerged as frequent systemic co-occurrence topics, while caries, ulcers, and tooth loss were represented in context of more generalized health decline. The publication-level clustering further revealed interdisciplinary and specialty-specific research domains. Together, these findings offer a structured, literature-level overview of the field and highlight both well-established and underexplored co-occurrence patterns—informing future research priorities, interdisciplinary collaboration, and public health strategies for comprehensive oral-systemic care.

## Supplementary Material

ooag130_Supplementary_Data

## Data Availability

The co-occurrence data underlying this study were derived from PubMed-indexed publications (1995-2025) accessed via the PubMed API. All publications are publicly available through PubMed (https://www.ncbi.nlm.nih.gov/pubmed). Search queries, co-occurrence matrices, and detailed clustering results are available from the corresponding author upon reasonable request.

## Code availability

All analysis code used in this study including scripts for PubMed API retrieval, co-occurrence matrix construction, hierarchical clustering, UMAP dimensionality reduction, K-means clustering, and visualization is publicly available on GitHub at https://github.com/BhumiAPatel/PubMed-Abstracts. The repository also contains the complete list of MeSH terms and keyword strings used for all condition queries, enabling full reproducibility of the reported analyses.
