# Supplementary material for: Mapping oral-systemic health relationships: a data-driven analysis of co-occurrence patterns in biomedical literature
Source: JAMIA Open. 2026 Jul 10;9(4):ooag130. doi: 10.1093/jamiaopen/ooag130 (PMC13354603; doi:10.1093/jamiaopen/ooag130)
Supplement: ooag130_Supplementary_Data [file ooag130_supplementary_data.docx]

**Supplementary Material**


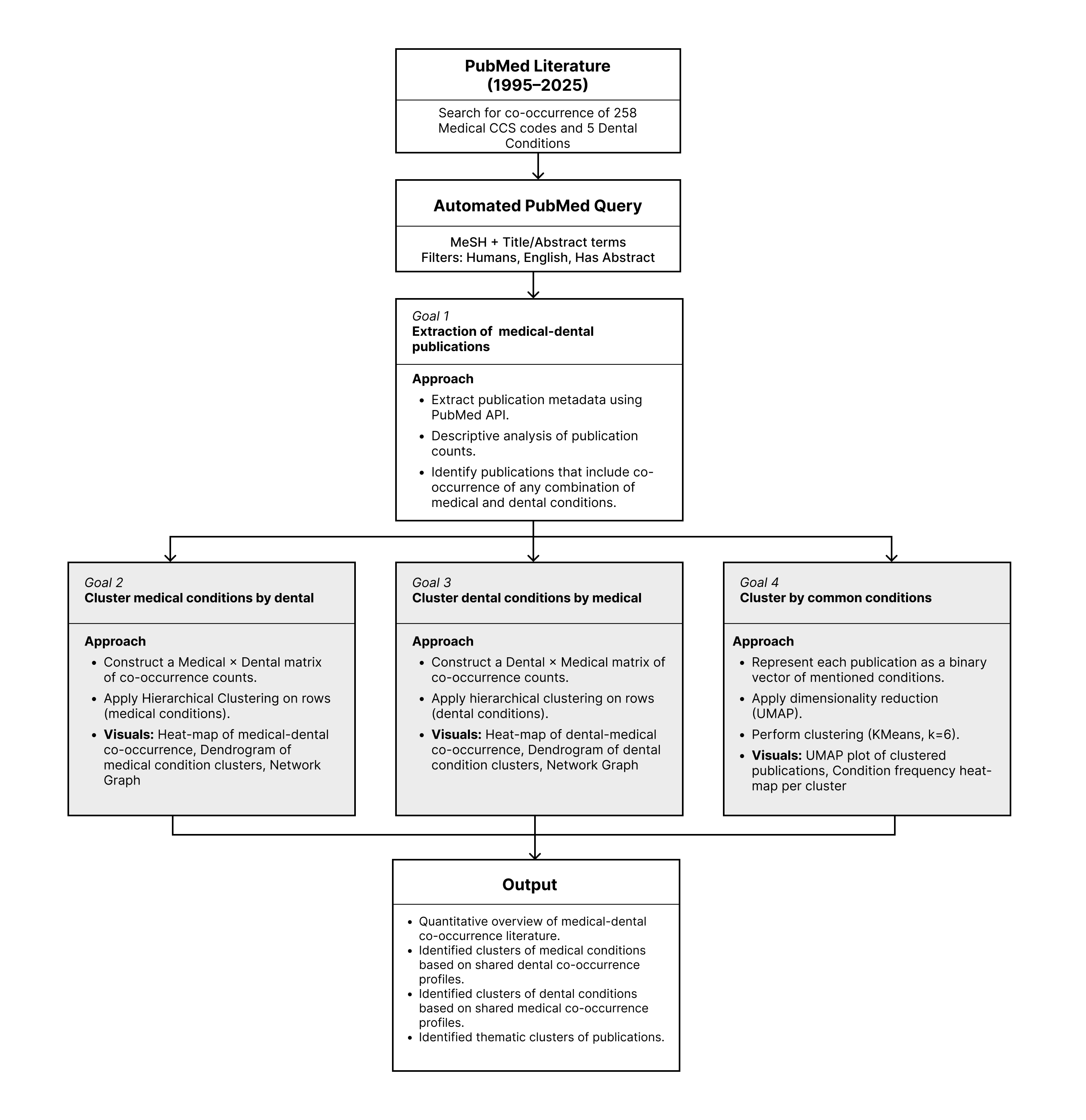


**Supp. Figure 1: Framework for Analyzing Oral-Systemic Co-occurrence in PubMed Literature.** Methodological framework illustrating the four-stage data-driven approach employed in this study to analyze co-occurrence patterns between systemic medical conditions and oral diseases in biomedical literature. The framework encompasses data retrieval from PubMed (1995-2025), automated search strategy combining 256 systemic conditions with 5 oral conditions, analytical methods including hierarchical and K-means clustering, and visualization approaches using dendrograms, heatmaps, and network graphs.


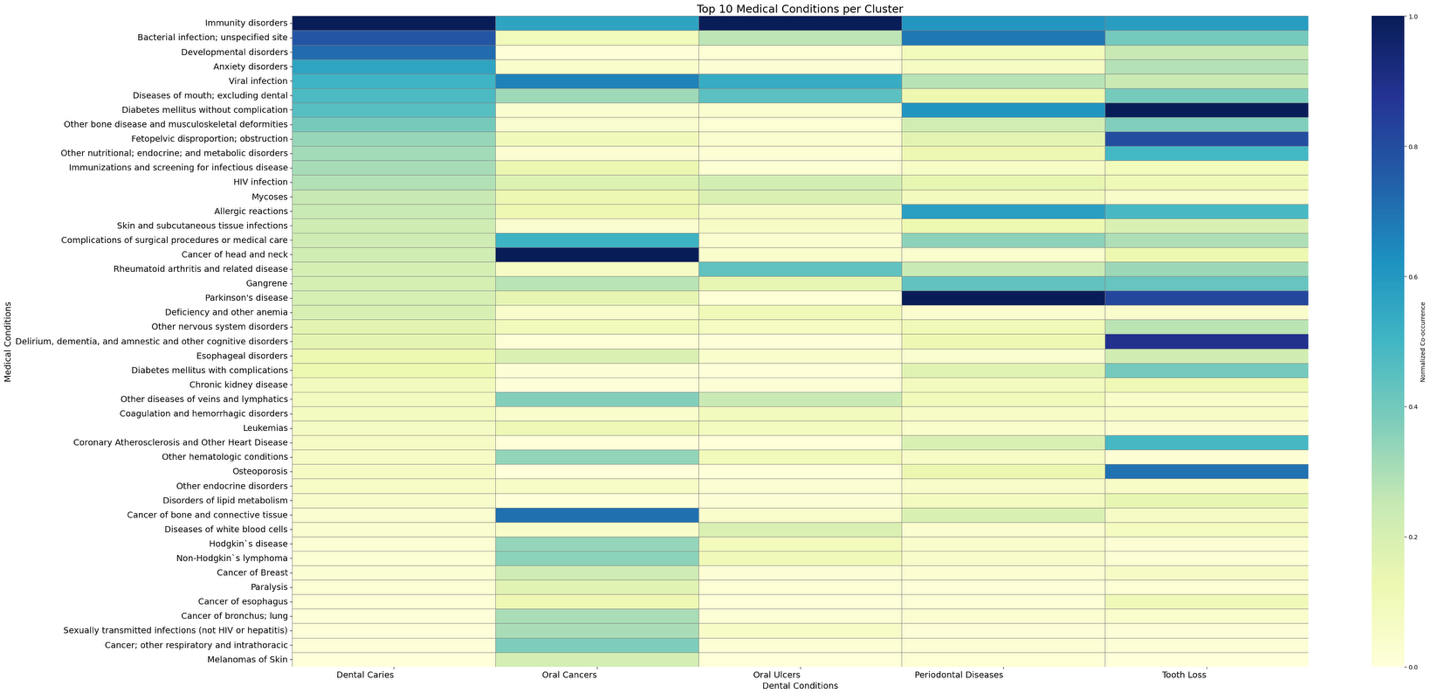


**Supp. Figure 2: Heat Map of Top Medical-Dental Conditions.** Heatmap illustrating normalized co-occurrence intensity between top systemic medical conditions (rows, grouped by eight clusters) and five oral health conditions (columns). Darker shades indicate stronger relative co-occurrence patterns, while lighter shades indicate weaker co-occurrence patterns. The heatmap is sorted by dental caries to emphasize differential co-occurrence patterns across oral health categories.


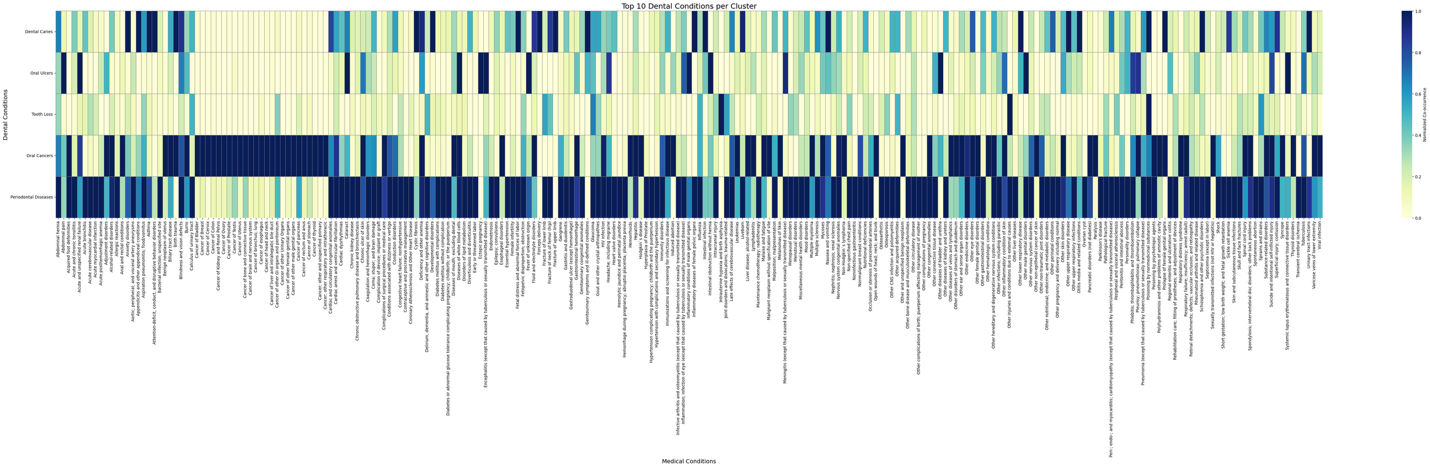


**Supp. Figure 3: Heat Map of Top Dental-Medical Conditions.** Heatmap displaying top systemic medical conditions (columns) associated with each of the three dental condition clusters (rows). Cell intensity indicates raw co-occurrence frequency counts, with darker shades representing higher frequencies. The visualization emphasizes differential systemic co-occurrence patterns across dental condition clusters, revealing distinct research concentrations for each dental condition grouping


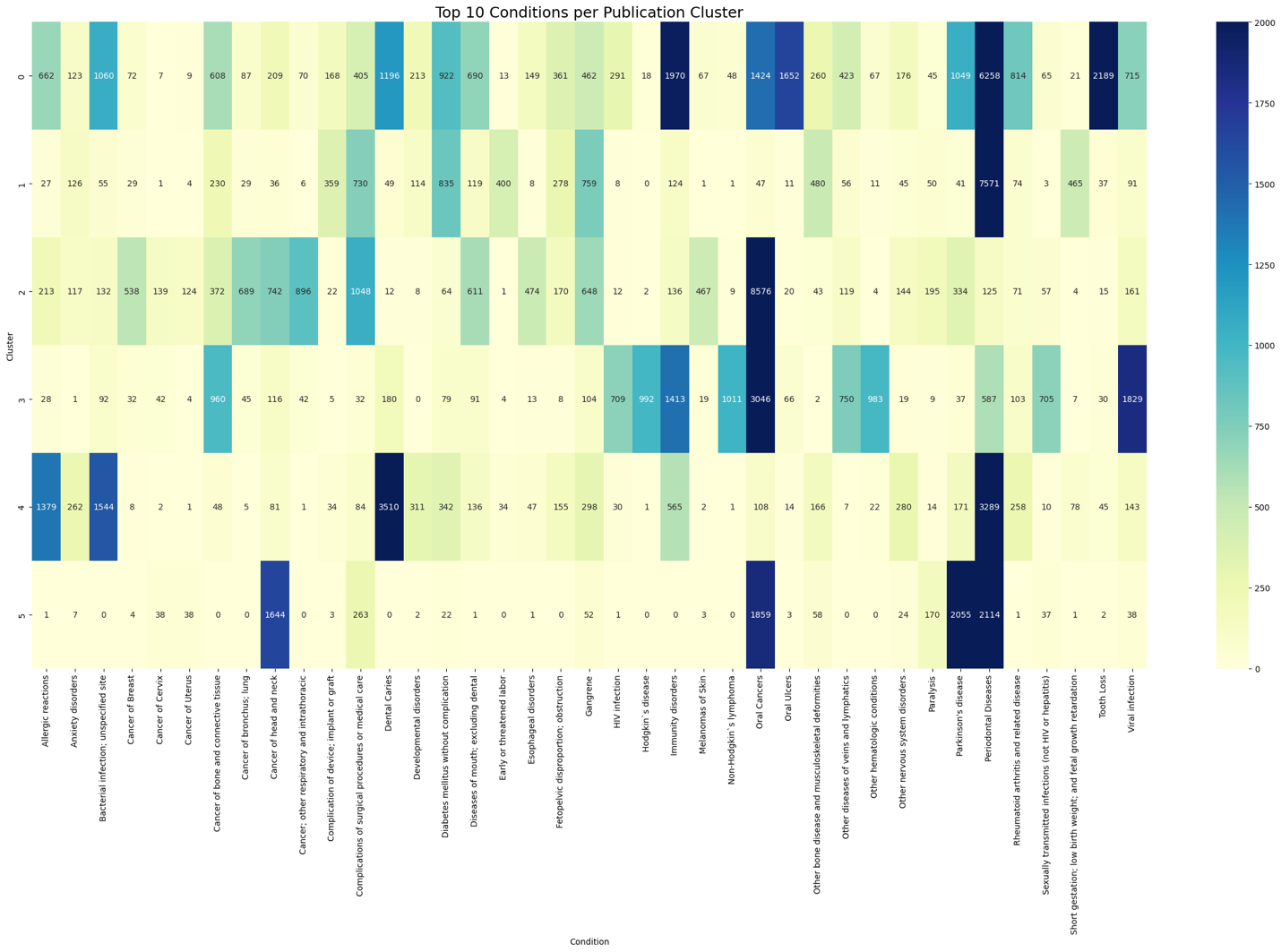


**Supp. Figure 4: Heat Map of Top Conditions Per Publication Clusters.** Heatmap presenting top medical and dental conditions (columns) most frequently mentioned within each of six publication clusters (rows). Each cluster exhibits distinct thematic concentration, with oncology and inflammation themes dominating the literature landscape. The visualization provides a quantitative profile of thematic composition for each publication grouping.

**Table 1: Top 10 Medical – Dental Co-occurrence Count**

| **Clusters** | **Medical Conditions** | **Co-occurrence Count (N)** | **Dental Condition** | **Co-occurrence Count (n)** |
| --- | --- | --- | --- | --- |
| **Cluster 1** | Parkinson’s disease | 3919 | Periodontal Diseases | 10693 |
|  | Bacterial infection; unspecified site | 3168 | Oral Cancers | 1780 |
|  | Diabetes mellitus without complication | 2566 | Dental Caries | 1049 |
|  | Allergic reactions | 2461 | Tooth Loss | 669 |
|  | Gangrene | 2433 | Oral Ulcers | 356 |
| **Cluster 2** | Viral infection | 3334 | Oral Cancers | 2751 |
|  | Diseases of mouth; excluding dental | 1904 | Periodontal Diseases | 2087 |
|  | Rheumatoid arthritis and related disease | 1430 | Oral Ulcers | 960 |
|  |  |  | Dental Caries | 665 |
|  |  |  | Tooth Loss | 205 |
| **Cluster 3** | Immunity disorders | 4791 | Periodontal Diseases | 1960 |
|  |  |  | Oral Cancers | 1467 |
|  |  |  | Oral Ulcers | 681 |
|  |  |  | Dental Caries | 558 |
|  |  |  | Tooth Loss | 125 |
| **Cluster 4** | Cancer of breast | 725 | Oral Cancers | 7844 |
|  | Melanomas of skin | 610 | Periodontal Diseases | 6925 |
|  | Leukemias | 550 | Dental Caries | 2215 |
|  | Paralysis | 489 | Oral Ulcers | 1437 |
|  | Coagulation and hemorrhagic disorders | 440 | Tooth Loss | 826 |
|  | Deficiency and other anemia | 422 |  |  |
|  | Other endocrine disorders | 385 |  |  |
|  | Cancer of esophagus | 385 |  |  |
|  | Chronic kidney disease | 360 |  |  |
|  | Disorders of lipid metabolism | 344 |  |  |
| **Cluster 5** | Fetopelvic disproportion; obstruction | 1157 | Periodontal Diseases | 1315 |
|  | Delirium, dementia, and amnestic and other cognitive disorders | 697 | Tooth Loss | 511 |
|  | Osteoporosis | 620 | Dental Caries | 310 |
|  |  |  | Oral Cancers | 302 |
|  |  |  | Oral Ulcers | 36 |
| **Cluster 6** | Other bone disease and musculoskeletal deformities | 1105 | Periodontal Diseases | 6696 |
|  | Other nervous system disorders | 809 | Dental Caries | 2697 |
|  | Developmental disorders | 778 | Oral Cancers | 2239 |
|  | Coronary Atherosclerosis and other heart disease | 775 | Tooth Loss | 1058 |
|  | Esophageal disorders | 764 | Oral Ulcers | 313 |
|  | Other nutritional; endocrine; and metabolic disorders | 762 |  |  |
|  | Diabetes mellitus with complications | 739 |  |  |
|  | Anxiety disorders | 738 |  |  |
|  | Immunization and screening for infectious disease | 731 |  |  |
|  | Skin and subcutaneous tissue infection | 712 |  |  |
| **Cluster 7** | Cancer of head and neck | 2919 | Oral Cancers | 5812 |
|  | Complications of surgical procedures or medical care | 2692 | Periodontal Diseases | 1879 |
|  | Cancer of bone and connective tissue | 2535 | Dental Caries | 271 |
|  |  |  | Tooth Loss | 104 |
|  |  |  | Oral Ulcers | 80 |
| **Cluster 8** | Other disease of veins and lymphatics | 1514 | Oral Cancers | 7221 |
|  | HIV infection | 1272 | Periodontal Diseases | 2002 |
|  | Other hematologic conditions | 1187 | Oral Ulcers | 1058 |
|  | Non-Hodgkin’s lymphoma | 1174 | Dental Caries | 455 |
|  | Hodgkin’s disease | 1102 | Tooth Loss | 105 |
|  | Cancer; other respiratory and intrathoracic | 1056 |  |  |
|  | Cancer of bronchus; lung | 914 |  |  |
|  | Sexually transmitted infections (not HIV or hepatitis) | 894 |  |  |
|  | Mycoses | 893 |  |  |
|  | Diseases of white blood cells | 420 |  |  |

**Table 2: Top 10 Dental – Medical Co-occurrence Count**

| **Clusters** | **Dental Conditions** | **Co-occurrence Count (N)** | **Medical Conditions** | **Co-occurrence Count (n)** |
| --- | --- | --- | --- | --- |
| **Cluster 1** | Dental Caries | 8220 | Immunity disorders | 1364 |
|  | Oral Ulcers | 4921 | Bacterial infection; unspecified site | 698 |
|  | Tooth Loss | 3703 | Viral infection | 695 |
|  |  |  | Diseases of mouth; excluding dental | 653 |
|  |  |  | Diabetes mellitus without complication | 494 |
|  |  |  | Rheumatoid arthritis and related disease | 482 |
|  |  |  | Developmental disorders | 465 |
|  |  |  | Anxiety disorders | 385 |
|  |  |  | Fetopelvic disproportion; obstruction | 383 |
|  |  |  | HIV infection | 330 |
| **Cluster 2** | Oral Cancers | 29416 | Cancer of head and neck | 2626 |
|  |  |  | Cancer of bone and connective tissue | 1848 |
|  |  |  | Viral infection | 1739 |
|  |  |  | Immunity disorders | 1467 |
|  |  |  | Complications of surgical procedures or medical care | 1338 |
|  |  |  | Cancer; other respiratory and intrathoracic | 985 |
|  |  |  | Other diseases of veins and lymphatics | 974 |
|  |  |  | Non-Hodgkin`s lymphoma | 929 |
|  |  |  | Other hematologic conditions | 900 |
|  |  |  | Hodgkin`s disease | 885 |
| **Cluster 3** | Periodontal Diseases | 33557 | Parkinson's disease | 3231 |
|  |  |  | Bacterial infection; unspecified site | 2218 |
|  |  |  | Diabetes mellitus without complication | 1978 |
|  |  |  | Immunity disorders | 1960 |
|  |  |  | Allergic reactions | 1865 |
|  |  |  | Gangrene | 1401 |
|  |  |  | Complications of surgical procedures or medical care | 1137 |
|  |  |  | Viral infection | 900 |
|  |  |  | Rheumatoid arthritis and related disease | 783 |
|  |  |  | Other bone disease and musculoskeletal deformities | 700 |

**Table 3: Top 10 Publications – Medical and Dental Conditions Co-occurrence Count**

| **Clusters** | **Medical and Dental Conditions** | **Co-occurrence Count (N)** |
| --- | --- | --- |
| **Cluster 0** | Periodontal Diseases | 6258 |
|  | Tooth Loss | 2189 |
|  | Immunity disorders | 1970 |
|  | Oral Ulcers | 1652 |
|  | Oral Cancers | 1424 |
|  | Dental Caries | 1196 |
|  | Bacterial infection; unspecified site | 1060 |
|  | Parkinson’s disease | 1049 |
|  | Diabetes mellitus without complication | 922 |
|  | Rheumatoid arthritis and related disease | 814 |
| **Cluster 1** | Periodontal Diseases | 7571 |
|  | Diabetes mellitus without complications | 835 |
|  | Gangrene | 759 |
|  | Complications of surgical procedures or medical care | 730 |
|  | Other bone disease and musculoskeletal deformities | 480 |
|  | Short gestation; low birth weight; and fetal growth retardation | 465 |
|  | Early or threatened labor | 400 |
|  | Complications of device; implant or graft | 359 |
|  | Fetopelvic disproportion; obstruction | 278 |
|  | Cancer of bone and connective tissue | 230 |
| **Cluster 2** | Oral Cancers | 8576 |
|  | Complications of surgical procedures or medical care | 1048 |
|  | Cancer; other respiratory and intrathoracic | 896 |
|  | Cancer of head and neck | 742 |
|  | Cancer of bronchus; lung | 689 |
|  | Gangrene | 648 |
|  | Disease of mouth; excluding dental | 611 |
|  | Cancer of breast | 538 |
|  | Esophageal disorders | 474 |
|  | Melanomas of skin | 467 |
| **Cluster 3** | Oral Cancers | 3046 |
|  | Viral infection | 1829 |
|  | Immunity disorders | 1413 |
|  | Non-Hodgkin`s lymphoma | 1011 |
|  | Hodgkin’s disease | 992 |
|  | Other hematologic conditions | 983 |
|  | Cancer of bone and connective tissue | 960 |
|  | Other diseases of veins and lymphatics | 750 |
|  | HIV infection | 709 |
|  | Sexually transmitted infections (not HIV or hepatitis) | 705 |
| **Cluster 4** | Dental Caries | 3510 |
|  | Periodontal Diseases | 3289 |
|  | Bacterial infection; unspecified site | 1544 |
|  | Allergic reactions | 1379 |
|  | Immunity disorders | 565 |
|  | Diabetes mellitus without complication | 342 |
|  | Developmental disorders | 311 |
|  | Gangrene | 298 |
|  | Other nervous system disorders | 280 |
|  | Anxiety disorders | 262 |
| **Cluster 5** | Periodontal Diseases | 2114 |
|  | Parkinson’s disease | 2055 |
|  | Oral Cancers | 1859 |
|  | Cancer of head and neck | 1644 |
|  | Complications of surgical procedures or medical care | 263 |
|  | Paralysis | 170 |
|  | Other bone disease and musculoskeletal deformities | 58 |
|  | Gangrene | 52 |
|  | Cancer of uterus | 38 |
|  | Cancer of cervix | 38 |
